# Comparative effects of high-intensity interval training and moderate-intensity continuous training on weight and metabolic health in college students with obesity

**DOI:** 10.1038/s41598-024-67331-z

**Published:** 2024-07-17

**Authors:** Xu Song, Xianyou Cui, Wenbo Su, Xueyan Shang, Meng Tao, Jing Wang, Chang Liu, Yaowei Sun, Hezhang Yun

**Affiliations:** 1 School of Physical Education, Zhejiang Guangsha Vocational and Technical University of Construction, Dongyang, China; 2 College of Sports Science, Qufu Normal University, Qufu, China; 3 Department of Sports Teaching and Research, Lanzhou University, Lanzhou, China; 4 Yufeng Experimental School, Kunshan, China; 5 School of Sport Science, Beijing Sport University, Beijing, China; 6 Moscow State Academy of Physical Education, Moscow, Russia; 7 School of Exercise and Health, Shanghai University of Sport, Shanghai, China

**Keywords:** High-intensity interval training, Moderate-intensity continuous training, College students, Body composition, Lipid profiles, Metabolic states, Health care, Weight management

## Abstract

The purpose of this study was to compare the effects of High-Intensity Interval Training (HIIT) and Moderate-Intensity Continuous Training (MICT) on weight, body composition, blood lipid indicators, and metabolic status in college students living with obesity. The study focused on a sample of 40 college students living with obesity, including 20 males and 20 females, aged between 18 and 25. Participants were randomly assigned to either the HIIT group or the MICT group. Both groups underwent an 8-week intervention, consisting of three sessions per week with alternate-day training. The MICT group's training consisted of continuous aerobic exercise for 35 min at 60–70% of maximum heart rate. The HIIT group engaged in 28 min of alternating high-intensity and low-intensity exercise, where the high-intensity phase was at 85–90% of maximum heart rate for 4 min, followed by a 3-min recovery period at 50–60% of maximum heart rate, repeated four times. Both groups underwent heart rate monitoring before and after the training sessions to ensure the accuracy of the training intensity. Within each group, further distinctions were made based on gender, resulting in the following subgroups: Male HIIT group (n = 10), Female HIIT group (n = 10), Male MICT group (n = 10), and Female MICT group (n = 10). Differences in anthropometric and biochemical indicators among the groups were analyzed, and the different effects of the two intervention strategies on the obese college student population were comprehensively evaluated. Compared to the baseline assessment, the HIIT group showed a more favorable declining trend than the MICT group in terms of body morphology and body composition, particularly in the aspect of body fat percentage (BF%). The male HIIT group, female HIIT group, male MICT group, and female MICT group respectively reduced by − 23.71%, − 26.76%, − 9.81%, − 7.16%. Male and female HIIT group experienced a more pronounced decrease compared to the MICT group, with the female HIIT group reducing an additional 3.75% more than the male HIIT group. Regarding intergroup differences, BF% significant differences were shown between male MICT group and the HIIT group (P < 0.05), and female MICT group and the HIIT group (P < 0.01). In terms of biochemical indicators, the HIIT group also presented a more favorable declining trend compared to the MICT group, with male HIIT participants showing more reduction than female HIIT participants, especially in total cholesterol (TC) (10.64%), low-density lipoprotein cholesterol (LDL-C) (11.73%), alanine aminotransferase (ALT) (11.99%), and uric acid (UA) (11.76%). Regarding triglycerides (TG), significant intergroup differences were observed between male MICT and HIIT groups (P < 0.01) and female MICT and HIIT groups (P < 0.01). Concerning ALT, a significant difference was shown between female MICT and HIIT groups (P < 0.01), while no significant difference was observed among male participants. Overall, for college students living with obesity, both HIIT and MICT have shown positive effects. Among these, HIIT demonstrates greater effectiveness compared to MICT in BF% and biochemical markers.

## Introduction

The statistics from the World Health Organization (WHO) have highlighted obesity as one of the most severe global public health threats to human well-being^1,2^. Over the past two decades, investigations and analyses of the physical condition of Chinese residents have indicated an escalating trend in the obesity rate among students^3,4^. As modern economies and societies rapidly progress and evolve, many individuals' daily behavioral patterns and dietary habits have undergone significant transformations. Factors such as improper dietary structures, irregular habits, prolonged sedentary behavior, insufficient physical activity, academic and occupational pressures, late nights, and irregular sleep patterns are closely linked to the emergence of obesity. Concurrently, obesity often results in lipid abnormalities that can gradually progress into a series of metabolic disorders, including hypertension, hyperlipidemia, atherosclerosis, and metabolic syndrome^5,6^. As vital constituents of the college students population, college students find themselves in a crucial phase of physical growth and development. This period is pivotal for cultivating robust physical health and establishing exercise habits, which significantly influence their physical and mental well-being, career planning, employment prospects, and future development^7^. Simultaneously, pertinent research has indicated a gradual decline in the physical fitness of college students, and various indicators of physical health have shown significant declines corresponding to the increase in obesity rates. These circumstances directly or indirectly underscore the adverse impacts of obesity on the development of college students^8^.

In recent years, HIIT and MICT have gained considerable research attention as two prominent exercise regimens, making them focal points in the intervention of college students living with obesity. HIIT involves alternating periods of high-intensity and low-intensity exercise to enhance cardiovascular function and raise metabolic rates. On the other hand, MICT involves relatively steady moderate-intensity aerobic exercise over longer durations to stimulate fat oxidation and cardiovascular health. These two training approaches exhibit distinct differences in exercise intensity, duration, and recovery time, thereby prompting research inquiries into their distinct effects on college students living with obesity. A substantial body of research has already examined the effects of HIIT and MICT on weight loss and health improvement in adults^8–12^. These studies indicate that both training methods have positive effects on enhancing aerobic capacity, promoting fat oxidation, and improving cardiovascular health. However, research comparing these two training methods, particularly in the context of college students, especially college students living with obesity, remains relatively limited.

This study is based on previous domestic and international research and aims to conduct an 8 week exercise intervention experiment using HIIT and MICT among college students living with obesity. Through statistical analysis of different exercise training methods, we aim to explore the impact of HIIT and MICT on the weight, body composition, blood lipid indicators, and metabolic status of college students living with obesity. This investigation seeks to determine whether there are differences in the effectiveness of these two exercise programs in improving the physical health of college students. Prior research has indicated^13^ that for overweight and/or obese adults, HIIT is superior to MICT in improving cardiopulmonary fitness. To enrich the research on the impact of HIIT on fat loss and lipid metabolism, enhance the theoretical contributions regarding its effects on fat loss and lipid metabolism, and provide experimental evidence for more efficient weight loss strategies and improved physical fitness for the obese population, we have designed this experimental study. The primary objective of this study is to compare the effects of HIIT and MICT interventions on anthropometric indicators, body composition, and biochemical indicators in college students living with obesity, and to investigate any potential differences in these effects based on gender.

## Materials and methods

### Subject recruitment

This study focuses on college students living with obesity as research subjects, aiming to investigate the effects of HIIT and MICT on the weight, body composition, lipid profiles, and metabolic states of obese college students. The inclusion criteria for participant recruitment were as follows: (1) aged between 18 and 25 years; (2) having a BMI ≥ 25; (3) maintaining stable weight for the past 2–3 months without significant fluctuations; (4) lacking medical history of metabolic, hormonal, orthopedic, or cardiovascular diseases, as well as infectious diseases, and not undergoing periodic prescription medication; (5) to avoid interference from other forms of exercise, participants are required not to engage in any additional physical activities beyond their normal daily routines (such as walking, climbing stairs) including but not limited to activities like running. The subjects are all full-time college students. Research indicates that the BMI cutoff for obesity in Asians is slightly lower than the value recommended by the World Health Organization, which is BMI < 25^4,14,15^. Therefore, we have set a BMI ≥ 25 as one of the criteria for participant selection. This study was approved by the Academic Research Ethics Committee of Qufu Normal University (Ethics Committee No. 2022079) and registered with the Chinese Clinical Trial Registry (ChiCTR) under registration number ChiCTR2300070895,26th April 2023. Before the experiment began, all participants completed a questionnaire using the long version of the International Physical Activity Questionnaire (IPAQ) to assess their daily physical activity levels and understand each individual's specific situation. Informed consent was obtained from all participants through the reading and signing of the informed consent form before formal participation in the study. The demographic information of the participants is presented in Table 1. There were no statistically significant differences in age, height, weight, Body Mass Index (BMI), Waist Circumference (WC), Hip Circumference (HC), Waist-to-Hip Ratio (WHR), and BF% between the two groups of males and females at baseline.

**Table 1:** Basic characteristics of subjects.

Group	Age/(year)	Height/(cm)	Mass/(kg)	BMI/(kg/m^2^)	WC/(cm)	HC/(cm)	WHR	BF%
Male HIIT (n = 10)	21.60 ± 1.84	173.40 ± 4.53	88.41 ± 3.50	29.43 ± 1.27	105.40 ± 7.56	109.35 ± 7.42	0.96 ± 0.0	27.93 ± 3.76
Female HIIT (n = 10)	21.90 ± 2.23	168.00 ± 5.25	82.04 ± 6.44	29.03 ± 1.07	90.70 ± 4.32	100.30 ± 4.11	0.90 ± 0.03	29.34 ± 2.47
Male MICT (n = 10)	21.00 ± 1.41	170.20 ± 10.78	86.82 ± 12.05	29.85 ± 1.59	105.50 ± 5.72	111.00 ± 3.89	0.95 ± 0.03	28.13 ± 2.90
Female MICT (n = 10)	22.20 ± 1.55	163.90 ± 4.41	79.56 ± 4.44	29.63 ± 1.48	90.00 ± 6.67	100.40 ± 5.32	0.90 ± 0.05	29.48 ± 4.21

### Sample size calculation

**Figure 1:**
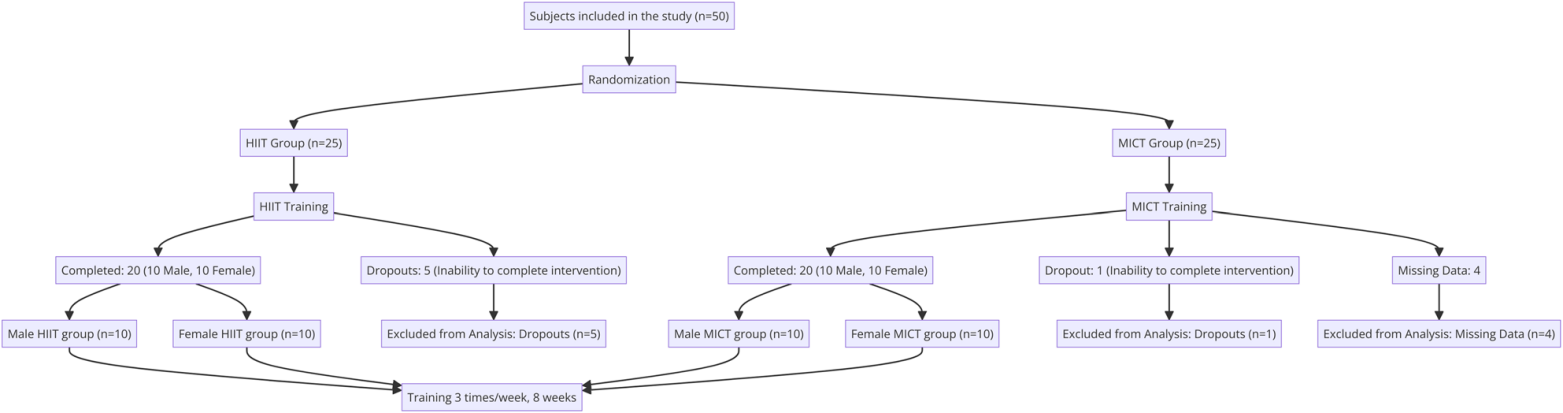
Experimental grouping diagram.

This study utilized G*Power 3.1 software, a widely used statistical tool for calculating the required sample size for experiments to achieve sufficient statistical power^16^. The parameters considered included a beta error of 20% (power = 0.80) and an alpha error of 5%. The reduction in BF% serves as a primary research indicator in this study, drawing insights from prior research on the impact of HIIT on BF%^17^. The effect size (ES) is set at 0.37, additionally, research indicates that an effect size of 0.2 is considered small, 0.5 is medium, and 0.8 is large^18,19^, with a total of four test groups (two groups for each gender) and two measurements. G*power calculations clearly indicate that the required sample size for each group should be 7. The experiment was conducted at Qufu Normal University. To expand the sample size, a total of 50 college students living with obesity (25 males and 25 females) were recruited as participants. Using a random number table, eligible male and female participants were randomly assigned to either the HIIT group or the MICT group. The HIIT group engaged only in high-intensity interval training, while the MICT group participated solely in moderate-intensity continuous training. During the course of the experiment, 6 participants could not complete the entire exercise intervention, and 4 participants had missing data, resulting in a final analysis of 40 valid participants. These comprised the male HIIT group (n = 10), female HIIT group (n = 10), male MICT group (n = 10), and female MICT group (n = 10). Refer to Fig. 1 for the detailed process.

### Training protocol

In this experiment, a power treadmill (Baodelong Fitness Co., LTD, JB-8800E, China) was utilized for training, with an intervention period lasting 8 weeks. Training sessions were scheduled three times per week on non-consecutive days, specifically in the evening between 19:00 and 21:00. To reduce individual variability, we conducted a preliminary test on the subjects' maximal heart rate (HRmax) before the experiment and measured the actual oxygen consumption of the participants at their target heart rate, thereby determining the training intensity for the subjects. The training protocols for each group were as follows:

HIIT Group: Prior to commencing the HIIT training, a 3-min warm-up at 35% of HRmax was conducted. Following warm-up, participants adjusted treadmill speed based on their heart rate to quickly reach 85–90% of HRmax and maintained this level for 4 min (heart rate should not fall below 85% of HRmax). This was followed by 3 min of exercise at 50–60% of HRmax. This cycle was repeated for a total of 4 sets, resulting in a training duration of 28 min. After the exercise, a cool-down and stretching routine was performed^20,21^. MICT Group: Similar to the HIIT group, the MICT group began with a 3-min warm-up at 35% of HRmax. After the warm-up, participants adjusted the treadmill speed to achieve a heart rate of 60–70% of HRmax. This heart rate level was sustained for 35 min (heart rate maintained between 60 and 70% of HRmax). The total training duration for the MICT group was 35 min. Following the exercise session, participants performed a cool-down and stretching routine. Throughout the entire exercise period for the participants, dedicated researchers were present to continuously encourage and remind the participants to reach their target heart rate range.

During the intervention, real-time heart rate monitoring was ensured by having both groups wear heart rate monitors (Polar H10, Finland) to adjust exercise intensity based on observed heart rate changes. One month before the experiment, we conducted a survey on the dietary habits and daily activities of the participants. We asked all participants to maintain their usual dietary habits, eating frequency, and preferences during the study period and to avoid any significant dietary changes during the intervention. Additionally, we provided general dietary advice, encouraging participants to maintain a balanced diet and avoid high-calorie, high-fat, and high-sugar foods. During the entire experimental period, we utilized the "WeChat" application to record the daily step count of the participants, allowing us to monitor their physical activity and ensure the accuracy and smooth progress of the experiment.

### Control of energy expenditure

To ensure that the energy expenditure was equivalent between the two groups during the intervention, participants in both groups followed the principle of expending the same amount of energy. This was achieved by testing the actual oxygen consumption levels of each participant at their target heart rate, thereby determining the training intensity for both groups. Specifically, the tests revealed that in the HIIT group, the average oxygen consumption was 1.596 L/min at 85–90% of maximum heart rate and 0.705 L/min at 50–60% of maximum heart rate. Therefore, based on the exercise regimen, the total oxygen consumption for the HIIT group was calculated as follows:

$${\text{Total Oxygen Consumption }}\left( {{\text{HIIT}}} \right) \, = {\text{ 4 sets }} \times \, \left( {{\text{4 min }} \times { 1}.{\text{596 L}}/{\text{min}}} \right) \, + {\text{ 4 sets }} \times \, \left( {{\text{3 min }} \times \, 0.{7}0{\text{5 L}}/{\text{min}}} \right) \, = { 33}.{\text{996 L}}.$$

For the MICT group, with an average oxygen consumption of 0.973 L/min at 60–70% of HRmax, the required exercise duration at the target heart rate was calculated as:

$${\text{Exercise Duration }}\left( {{\text{MICT}}} \right) \, = {\text{ Total Oxygen Consumption }}\left( {{\text{HIIT}}} \right) \, /{\text{ Oxygen Consumption Rate }}\left( {{\text{MICT}}} \right) \, = { 33}.{\text{996 L }}/ \, 0.{\text{973 L}}/{\text{min }} = { 34}.{\text{94 min}}.$$

As participants' exercise capacity improved, the exercise speed at the same target heart rate would naturally increase while maintaining consistent oxygen consumption levels for both groups.

### Rating of perceived exertion (RPE) assessment

In this experiment, the monitoring of exercise intensity utilized a combination of real-time heart rate and the RPE scale. To accurately gauge participants' fatigue levels during exercise, participants were thoroughly briefed on the scoring and significance of the RPE scale before the intervention. During the intervention, participants were adeptly guided to rate their perceived exertion using the RPE scale^22^. RPE values were collected from participants every 5 min. Based on the grading of exercise intensity established by the American College of Sports Medicine (ACSM) Table 2, the target heart rate zones were determined^23^. The intervention's intensity was determined by recording participants' subjective perception of exertion and real-time heart rate during exercise.

**Table 2:** ACMS exercise intensity grading and subjective physical sensory rating scale.

Intensity level	Maximum HR (%)	RPE value	Perceived exertion	Corresponding HR
Very light	< 35	< 10	Very light	< 69
Light	35–64	10–11	Light	70–106
Moderate	55–69	12–13	Moderate	107–136
Hard	70–89	14–16	Hard	137–175
Very hard	≥ 90	17–19	Very hard	≥ 176
Maximal	100	20	Maximal	197

### Measurement indicators

#### Body morphology and body composition testing

The following measurements were conducted to assess body morphology and body composition:

WC: measured using a caliper (DELI, China), the point of measurement was the midpoint between the lowest rib of the 12th pair and the upper edge of the iliac crest. During measurement, participants were asked to relax their abdomen, and the measurement personnel's line of sight was aligned with the caliper scale. HC: during HC measurement, participants were required to stand with their legs close together and straight, arms hanging naturally. The measurement personnel positioned the caliper at the pubic symphysis in the front and at the most prominent part of the gluteus maximus in the back. The measurement was taken three times, and the average value was recorded to the nearest 0.1 cm. Participants were instructed to minimize clothing interference during measurements. WHR: calculated by dividing the WC by the HC^24^. Height and Weight: Measured using an HGM-700 ultrasonic medical height and weight scale. Height was recorded in centimeters (cm) to the nearest 0.1 cm, and weight was recorded in kilograms (kg) to the nearest 0.1 kg. To minimize errors, three measurements were taken, and the average was recorded. Body fat percentage (BF%): measured using bioelectrical impedance analysis Inbody770 (Biospace Co. Ltd., Korea)^25^. Participants were required to fast for at least 8 h before the measurement, avoid vigorous exercise and excessive water intake, and remove any metallic objects during the measurement to ensure accuracy and a stable hydration state. The testing time was uniformly scheduled between 8:00 AM and 10:00 AM to minimize the impact of daily activities on the measurement results. Furthermore, it was ensured that participants were in a relaxed state during the measurement, and the same measurement procedures and technical standards were followed to reduce errors.

#### Blood collection

Subjects were uniformly positioned in a seated posture for venous blood collection, with instructions to sit sideways, ensuring their upper body was perpendicular to the ground. Their arm was placed on a stable operating table, the elbow resting on a cushion to maintain a straight line between the upper arm and forearm, with the palm slightly lower than the elbow, fully exposing the blood collection site. Blood was collected from the antecubital vein. Venous blood samples were uniformly collected between 8:00 AM and 10:00 AM. Participants were instructed to fast before blood collection, consuming a light meal the previous evening while avoiding high-sugar and high-fat foods. The fasting period before blood collection ranged between 9 and 12 h. Blood was collected using blood collection tubes containing Heparin as an anticoagulant. After collection, the tubes were gently shaken five times for thorough mixing, and then the samples were placed on ice and refrigerated. After collecting blood from all participants, the blood collection tubes were centrifuged at 3000 r/min and 4 °C for 10 min to separate the plasma^26^. The Architect C-8000 (Abbott Laboratories, USA) automated chemistry analyzer was utilized to measure total cholesterol (TC), triglycerides (TG), low-density lipoprotein cholesterol (LDL-C), high-density lipoprotein cholesterol (HDL-C), alanine aminotransferase (ALT), and uric acid (UA)^27^.

### Statistics

The experimental data were statistically processed using SPSS 22.0 software for statistical analysis, and GraphPad Prism 9.0 was utilized for data visualization. The results of various indicators for each participant were presented as mean ± standard deviation. Concerning the study's outcomes, we initially conducted a One-sample Kolmogorov–Smirnov test to ascertain normal data distribution. Subsequently, the data underwent a mixed ANOVA with a 2 × 2 × 2 structure (time × grouping × gender), the calculation of ηp2 uses the following categorization criteria: 0.10 indicates a small effect, 0.25 indicates a medium effect, and 0.40 indicates a large effect^28,29^. The time variable (pre-test and post-test) was employed as an intragroup factor, whereas the grouping (MICT and HIIT) and gender (male and female) served as intergroup factors. The dependent variables included outcome indicators such as weight, BMI, BF%, WC, HC, WHR, TC, TG, HDL-C, LDL-C, ALT, and UA. We scrutinized the outcome indicators for main effects, interactions, and simple effects across different groupings, genders, and before/after testing. Significance levels were denoted as follows: P < 0.05 indicates significant differences, P < 0.01 denotes highly significant differences, while P < 0.001 signifies extremely significant differences, and P > 0.05 suggests a lack of significant differences.

### Institutional review board statement

The study was conducted in accordance with the Declaration of Helsinki, and approved by the Institutional Review Board of the Academic Research Ethics Committee of Qufu Normal University (Ethics Committee No.2022079) and registered with the Chinese Clinical Trial Registry (ChiCTR) under registration number ChiCTR2300070895, 26th April 2023.

### Informed consent statement

Informed consent was obtained from all subjects involved in the study.

## Results

### Effects of different training modes on participants' body morphology indicators

#### Effects on weight, BMI, and BF%

**Figure 2:**
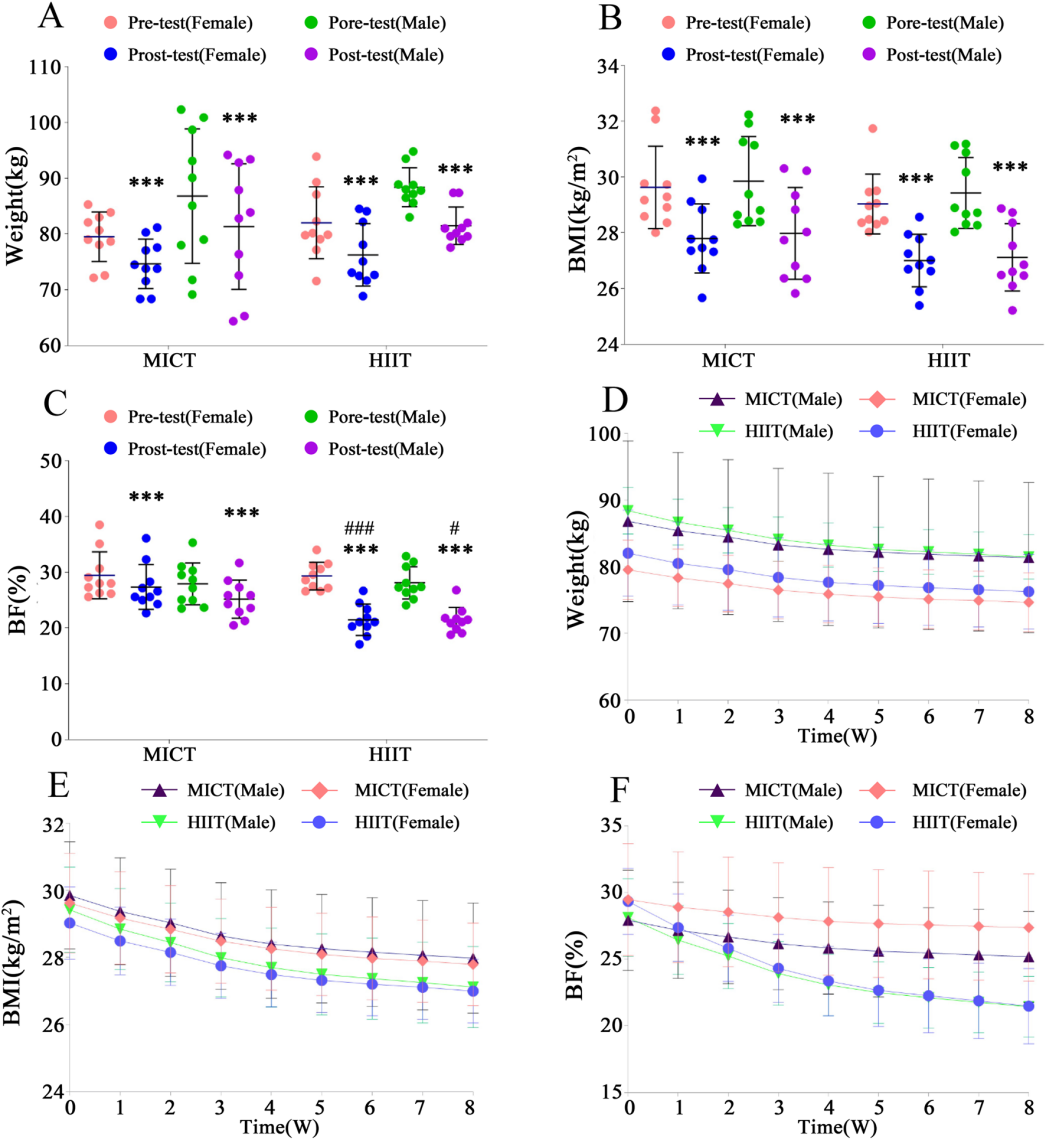
Effects of Different Exercises on Participants' Weight, BMI, and BF%. ***Denotes extremely significant differences from pretest values in Weight, BMI, and BF% (P < 0.001). ^###^Represents extremely significant differences between the HIIT and MICT groups (P < 0.001). # Represents significant differences between the HIIT and MICT groups (P < 0.05). *W* weeks.

To analyze the impact of different groups, genders, and pre-post tests on weight, BMI, and BF%, a mixed ANOVA was conducted. The improvement in the effects on weight, BMI, and BF% is depicted in Fig. 2A–C, showing highly significant differences for both male and female participants in the MICT and HIIT groups compared to before exercise (p < 0.001). In the MICT group, female participants exhibited significant average reductions in weight (4.89 ± 1.82 kg, [F = 72.26, P < 0.001, ηp^2^ = 0.67]), BMI (1.81 ± 0.71 kg/m^2^, [F = 84.93, P < 0.001, ηp^2^ = 0.70]), and BF% (2.11 ± 0.90, [F = 19.18, P < 0.001, ηp^2^ = 0.35]). Male participants in the MICT group also showed significant average reductions in weight (5.45 ± 2.18 kg, [F = 89.75, P < 0.001, ηp^2^ = 0.71]), BMI (1.87 ± 0.69 kg/m^2^, [F = 88.49, P < 0.001, ηp^2^ = 0.71]), and BF% (2.74 ± 1.10, [F = 32.35, P < 0.001, ηp^2^ = 0.47]). Similarly, in the HIIT group, female participants demonstrated significant average reductions in weight (5.75 ± 2.01 kg, [F = 99.91, P < 0.001, ηp^2^ = 0.74]), BMI (2.03 ± 0.68 kg/m^2^, [F = 104.51, P < 0.001, ηp^2^ = 0.74]), and BF% (7.85 ± 1.80, [F = 265.49, P < 0.001, ηp^2^ = 0.88]). Male participants in the HIIT group also exhibited significant average reductions in weight (6.92 ± 1.08 kg, [F = 144.70, P < 0.001, ηp^2^ = 0.80]), BMI (2.30 ± 0.36 kg/m^2^, [F = 134.51, P < 0.001, ηp^2^ = 0.79]), and BF% (6.67 ± 2.00, [F = 191.67, P < 0.001, ηp^2^ = 0.84]).

In the participants, both male (F = 0.17, P = 0.90, ηp^2^ = 0.00) and female (F = 0.01, P = 0.93, ηp^2^ = 0.00), no statistically significant differences were observed in the intergroup comparison of BF% between the MICT and HIIT groups at baseline. However, in the tests conducted after 8 weeks of divergent training, significant differences were noted between the MICT and HIIT groups for both males (F = 6.81, P < 0.05, ηp^2^ = 0.16) and females (F = 16.93, P < 0.001, ηp^2^ = 0.32). Specifically, the average body fat percentage in the female HIIT group decreased by 21.48% compared to the female MICT group post-training, while in the male HIIT group, there was a 14.81% reduction compared to the male MICT group. These findings indicate that HIIT significantly reduces body fat in both males and females, particularly demonstrating a notable improvement in females.

Figure 2D–F illustrates the changes in weight, BMI, and BF% for both males and females after 8 weeks of MICT and HIIT training. In both male and female participants from both groups, there is a noticeable downward trend in the mentioned indicators after the onset of exercise intervention, as depicted in Fig. 2D–F. In the MICT group, for female participants, there was a weight reduction of (− 3.10%) in the first 4 weeks and (− 1.06%) in the subsequent 4 weeks. Similarly, the BMI decreased by (− 3.12%) in the first four weeks and (− 1.06%) in the latter four weeks, while BF% showed a decrease of (− 3.67%) in the first four weeks and (− 1.12%) in the subsequent four weeks. For male participants in the MICT group, there was a weight reduction of (− 3.31%) in the first four weeks and (− 1.00%) in the subsequent four weeks. The BMI decreased by (− 3.33%) in the first four weeks and (− 1.00%) in the latter four weeks, while BF% showed a decrease of (− 4.93%) in the first four weeks and (− 1.64%) in the subsequent four weeks. In the HIIT group, for female participants, there was a weight reduction of (− 3.54%) in the first four weeks and (− 1.18%) in the subsequent four weeks. The BMI decreased by (− 3.52%) in the first four weeks and (− 1.17%) in the latter four weeks, while BF% showed a remarkable decrease of (− 14.61%) in the first four weeks and (− 5.21%) in the subsequent four weeks. For male participants in the HIIT group, there was a weight reduction of (− 3.98%) in the first four weeks and (− 1.38%) in the subsequent four weeks. The BMI decreased by (− 3.97%) in the first four weeks and (− 1.38%) in the latter four weeks, while BF% showed a decrease of (− 12.71%) in the first four weeks and (− 4.66%) in the subsequent four weeks.

The rate of decline in the first 4 weeks of the curve changes in weight, BMI, and BF% is significantly higher than the subsequent 4 weeks for both exercise regimens. Furthermore, the HIIT group exhibited a more favorable declining trend compared to the MICT group. Notably, in terms of BF%, both male and female participants in the HIIT group experienced a more pronounced reduction compared to the MICT group, with the most significant reduction observed in female participants in the HIIT group.

#### Effects of different training methods on WC, HC, and WHR

**Figure 3:**
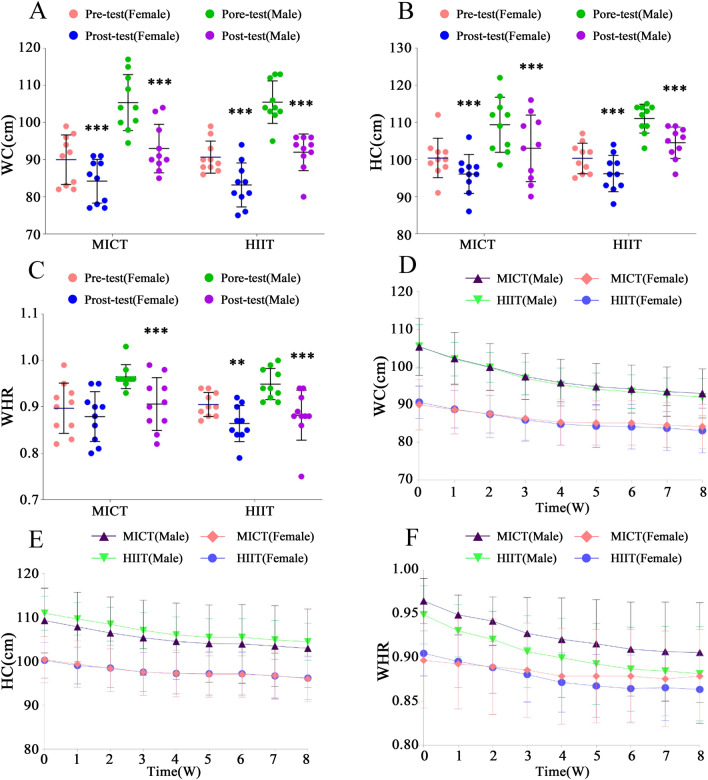
Effects of Different Exercise Modes on Participants' WC, HC, and WHR. ***Denotes extremely significant differences from pretest values in WC, HC, and WHR (P < 0.001). **Denotes highly significant differences from pretest values in WC, HC, and WHR (P < 0.01). *W* weeks.

For the improvement effects on WC, HC, and WHR, as depicted in Fig. 3A–C. In the MICT group: female participants exhibited a significant average reduction in WC, HC, and WHR of 5.79 ± 1.32 cm (F = 17.03, P < 0.001, ηp^2^ = 0.32), 4.30 ± 1.34 cm (F = 46.70, P < 0.001, ηp^2^ = 0.57), and 0.02 ± 0.02 cm (F = 1.49, P > 0.05, ηp^2^ = 0.04), respectively. In the HIIT group: female participants demonstrated a significant average reduction in WC, HC, and WHR of 7.50 ± 2.42 cm (F = 28.58, P < 0.001, ηp^2^ = 0.44), 4.10 ± 1.73 cm (F = 42.46, P < 0.001, ηp^2^ = 0.54), and 0.04 ± 0.02 cm (F = 7.70, P < 0.01, ηp^2^ = 0.18), respectively. In the MICT group: Male participants experienced a significant average reduction in WC, HC, and WHR of 12.40 ± 5.68 cm (F = 78.11, P < 0.001, ηp^2^ = 0.69), 6.35 ± 3.22 cm (F = 101.85, P < 0.001, ηp^2^ = 0.74), and 0.06 ± 0.07 cm (F = 15.95, P < 0.001, ηp^2^ = 0.31), respectively. In the HIIT group: Male participants demonstrated a significant average reduction in WC, HC, and WHR of 13.50 ± 6.24 cm (F = 92.58, P < 0.001, ηp^2^ = 0.72), 6.50 ± 0.85 cm (F = 106.72, P < 0.001, ηp^2^ = 0.75), and 0.07 ± 0.06 cm (F = 20.57, P < 0.001, ηp^2^ = 0.36), respectively.

It is evident that both interventions have highly significant effects on reducing WC and HC for both male and female participants. However, in terms of the reduction in WHR, the HIIT group exhibits a more pronounced effect compared to the MICT group. The changes in WC, HC, and WHR after 8 weeks are evident in Fig. 3D–F. For female participants in the MICT group, the reduction effects in the first four weeks were significantly greater than in the subsequent four weeks. In the first four weeks, there was a reduction in WC of (− 3.72%), HC of (− 2.11%), and WHR of (− 1.61%). In the following four weeks, the reduction was observed in WC of (− 0.63%), HC of (− 1.03%), and WHR of (− 0.63%). For male participants in the MICT group, there was a substantial reduction in WC of (− 6.26%), HC of (− 3.06%), and WHR of (-3.11%) in the first four weeks, followed by a decrease in WC of (− 1.90%), HC of (− 1.06%), and WHR of (− 0.82%) in the subsequent 4 weeks. In the HIIT group, for female participants, the first 4 weeks showed a reduction in WC of (− 4.39%), HC of (− 1.82%), and WHR of (− 2.63%). The following 4 weeks led to a reduction in WC of (− 1.42%), HC of (− 1.03%), and WHR of (− 0.41%). For male participants in the HIIT group, the first 4 weeks exhibited a reduction in WC of (− 6.66%), HC of (− 3.28%), and WHR of (− 3.42%), followed by a decrease in WC of (− 2.23%), HC of (− 0.95%), and WHR of (− 1.28%).

In the first four weeks of the WC, HC, and WHR curves, the rate of decline for both exercise regimens is significantly higher than the subsequent four weeks. Furthermore, compared to the MICT group, the HIIT group exhibits a more favorable declining trend.

### Effects of different training methods on participants' biochemical parameters

#### Effects of different training methods on participants' blood lipids profiles

**Figure 4:**
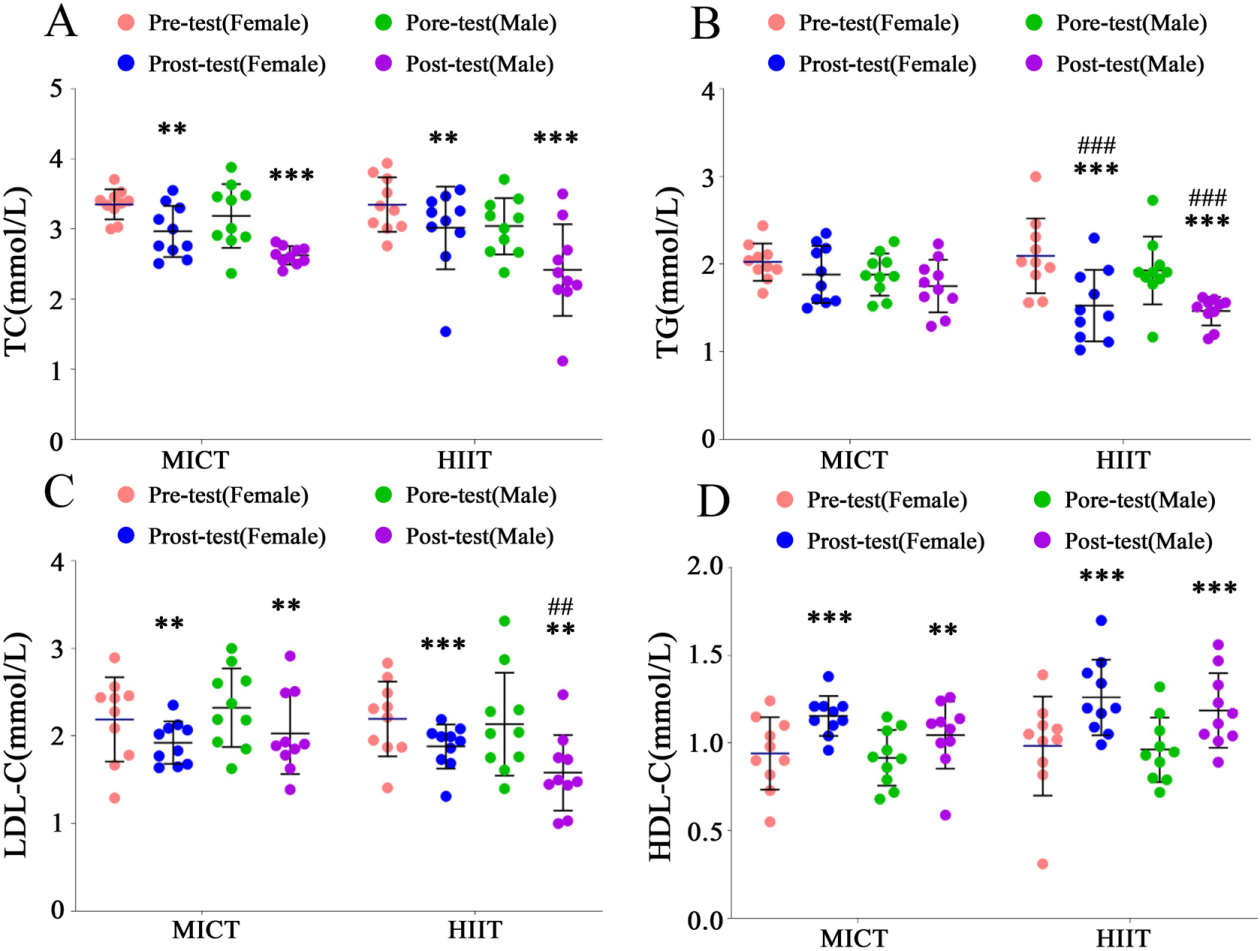
Effects of Different Exercise Modalities on Participants' Blood Lipids Profiles. ***Denotes extremely significant differences from pretest values in TC, TG, LDL-C and HDL-C (P < 0.001). **Denotes highly significant differences from pretest values in TC, TG, LDL-C and HDL-C (P < 0.01). ^###^Represents extremely significant differences between the HIIT and MICT groups (P < 0.001). ^##^Represents highly significant differences between the HIIT and MICT groups (P < 0.01).

The improvement effects on lipid profiles are evident in Fig. 4A–C. Compared to the baseline, female participants in the MICT group showed significant decreases in TC [F = 12.51, P < 0.01, ηp^2^ = 0.26], TG [F = 2.86, P > 0.05, ηp^2^ = 0.07], and LDL-C [F = 8.04, P < 0.01, ηp^2^ = 0.18]. Similarly, female participants in the HIIT group exhibited reductions in TC [F = 9.26, P < 0.01, ηp^2^ = 0.21], TG [F = 46.59, P < 0.001, ηp^2^ = 0.56], and LDL-C [F = 11.13, P < 0.01, ηp^2^ = 0.24].

For male participants, the improvement trends in TC, TG, and LDL-C were similar to those observed in females. Notably, the MICT group for males showed decreases in TC [F = 26.62, P < 0.001, ηp^2^ = 0.43], TG [F = 2.47, P > 0.05, ηp^2^ = 0.06], and LDL-C [F = 8.04, P < 0.01, ηp^2^ = 0.21]. Meanwhile, the HIIT group for males exhibited reductions in TC [F = 32.80, P < 0.001, ηp^2^ = 0.48], TG [F = 30.98, P < 0.001, ηp^2^ = 0.46], and LDL-C [F = 35.00, P < 0.001, ηp^2^ = 0.49].

In terms of TG, no statistically significant differences were observed between the MICT and HIIT groups at baseline for both females [F = 2.86, P > 0.05, ηp^2^ = 0.07] and males [F = 2.47, P > 0.05, ηp^2^ = 0.06]. However, following 8 weeks of training with different exercise modalities, significant differences were noted between the MICT and HIIT groups for both females [F = 46.59, P < 0.001, ηp^2^ = 0.56] and males [F = 30.98, P < 0.001, ηp^2^ = 0.46]. Specifically, the average reduction in TG post-training was 18.91% in the female HIIT group compared to the female MICT group, and 16.39% in the male HIIT group compared to the male MICT group. These results indicate that HIIT is significantly effective in reducing TG in both males and females, particularly showing notable improvement in females.

Regarding LDL-C, at baseline, no statistically significant differences were identified between the MICT and HIIT groups for either females (F = 0.00, P > 0.05, ηp^2^ = 0.00) or males (F = 0.74, P > 0.05, ηp^2^ = 0.02). However, after 8 weeks of exercise training involving different modalities, a significant difference was observed between the MICT and HIIT groups in males (F = 7.73, P < 0.01, ηp^2^ = 0.18), with the male HIIT group experiencing a 22.13% reduction in the average value of LDL-C compared to the male MICT group post-training. This indicates that HIIT has a significant effect on improving LDL-C in males.

The improvement effects on HDL-C are depicted in Fig. 4D. Compared to the baseline, female participants in the MICT group exhibited a significant improvement in HDL-C [F = 23.20, P < 0.001, ηp^2^ = 0.39], and similarly, female participants in the HIIT group also showed a significant improvement [F = 38.60, P < 0.001, ηp^2^ = 0.52]. Male participants in the MICT group demonstrated an improvement in HDL-C [F = 8.56, P < 0.01, ηp^2^ = 0.19], and male participants in the HIIT group also exhibited a significant improvement [F = 25.42, P < 0.001, ηp^2^ = 0.41]. However, there was no significant difference in HDL-C between the two groups.

#### Effects of different training modalities on participants' ALT and UA

**Figure 5:**
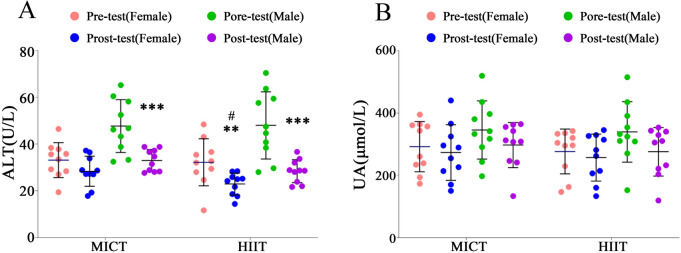
Effects of different exercise modalities on participants' ALT and UA. ***Denotes extremely significant differences from pretest values in ALT (P < 0.001). **Denotes highly significant differences from pretest values in ALT (P < 0.01). ^#^Represents significant differences between the HIIT and MICT groups (P < 0.05).

The changes in ALT and UA for both MICT and HIIT groups are illustrated in Fig. 5. Figure 5A illustrates the changes in ALT for both the MICT and HIIT groups before and after the intervention. In comparison to the pre-test, females in the MICT group showed no significant difference [F = 2.69, P > 0.05, ηp^2^ = 0.07], while females in the HIIT group exhibited a significant decrease [F = 10.12, P < 0.01, ηp^2^ = 0.22]. For males, both the MICT [F = 25.35, P < 0.001, ηp^2^ = 0.41] and HIIT [F = 45.16, P < 0.001, ηp^2^ = 0.56] groups demonstrated a significant reduction. There was no statistically significant difference between the female groups at baseline [F = 0.00, P > 0.05, P ηp^2^ = 0.00], but a significant difference emerged in the post-test [F = 5.44, P < 0.05, ηp^2^ = 0.13].

As for the improvement in uric acid (UA), Fig. 5B indicates that post-intervention measurements for both male and female participants in both groups exhibited a decreasing trend compared to the pre-test, but these changes were not statistically significant (P > 0.05).

## Discussion

Through a comprehensive tracking observation of the weekly variations in body weight, BMI, WC, HC, WHR, and BF% in both the HIIT and MICT groups, notable improvements were observed in all these indicators. This signifies that both HIIT and MICT interventions led to significant improvements in the body shape of college students living with obesity. However, in the tests conducted after 8 weeks of training utilizing different modalities, the female HIIT group exhibited a significant reduction in average BF% by 21.48% compared to the female MICT group, with a highly significant difference between the two groups (P < 0.001). The male HIIT group also demonstrated a noteworthy reduction in BF% by 14.81% compared to the male MICT group, showing a significant difference (P < 0.05). These results indicate that HIIT significantly reduces body fat in both males and females, particularly showing substantial improvements in female body fat. Overall, HIIT demonstrates superior weight reduction effects compared to MICT, aligning with findings from previous research^13,30–32^, particularly in terms of improvements in BF%. Consistent results were also observed by Miguet et al.^32^ in the enhancement of BF%. However, HIIT requires less time per training session than MICT, reducing the time cost. Additionally, Maturana et al.^33^ compared the cardiovascular responses between HIIT and MICT, indicating that for specific cardiovascular and cardiorespiratory enhancements, HIIT may offer more benefits than MICT, especially for individuals who were previously sedentary. The underlying reasons for these outcomes could be attributed to the substantial oxygen consumption generated by participants during exercise training. The heightened consumption of fat through increased respiration contributes to the reduction in BF%. Furthermore, the occurrence of Excess Post-Exercise Oxygen Consumption (EPOC), particularly evident in the female participants of the HIIT group, resulted in a significantly greater reduction in BF% compared to the females in the MICT group (P < 0.001). This could be due to the higher respiratory rate induced by HIIT, leading to greater fat consumption. The post-exercise recovery phase requires increased oxygen intake, further amplifying energy expenditure. HIIT has been demonstrated to increase total energy expenditure, exercise energy consumption, and EPOC, as opposed to bed rest^34^. In college students living with obesity, females might exhibit greater sensitivity to the fat-reducing effects of HIIT, possibly due to individual physiological variations. A significant observation is that the decline in the aforementioned indicators during the initial 4 weeks was more pronounced compared to the subsequent 4 weeks. This implies that the initial training phase has a more pronounced impact on body shape improvement, whereas the effects might gradually stabilize over time. Furthermore, muscle mass experienced growth under the influence of exercise intervention, with more intensive workouts leading to enhanced contraction of muscle fibers. This suggests that intense exercise might be more beneficial in improving skeletal muscle quality. Research suggests that HIIT can augment the synthesis rate of myofibrillar protein fibers^35^ as well as muscle cross-sectional area^36^. As a result, after 8 weeks of HIIT, a significant reduction in body fat content was observed among participants. However, due to the concurrent increase in muscle mass under the training intervention, the reduction in body weight and BMI was relatively moderate. Additionally, after 8 weeks of exercise training, both male and female participants exhibited a decrease in WHR. However, these values still exceeded the average levels of WHR among normal Asian males and females, indicating that the participants still predominantly fell under the category of central obesity. Further exercises and interventions are necessary to achieve more significant fat reduction effects. Additionally, HIIT surpasses MICT in promoting the secretion of catecholamines, adrenaline, norepinephrine, and growth hormone, which facilitate fat breakdown, thereby achieving effective weight loss^13^. In contrast to exercise intensity, the overall energy expenditure plays a more critical role in weight reduction. While MICT over a more extended period can lead to a substantial total energy expenditure for weight loss, HIIT achieves significant energy expenditure in a shorter duration.

Dyslipidemia refers to an abnormal lipid profile in the bloodstream, with hyperlipidemia being a major form of lipid abnormality. This condition is considered a pathogenic risk factor for cardiovascular diseases, characterized by elevated levels of TC, LDL-C and TG, along with decreased levels of HDL-C^37^. Studies have indicated that an increase in body TG concentration is associated with elevated levels of UA in obese individuals^38^. Furthermore, changes in glucose homeostasis may be linked to obesity and type 2 diabetes^38^. Elevated levels of ALT are associated with metabolic syndrome and obesity. Additionally, ALT serves as a crucial predictive indicator for diabetes. Importantly, it has been reported that ALT levels in obese individuals are higher than those in normal individuals^39^. Based on the results of biochemical parameter analysis in this study, both the HIIT and MICT groups exhibited an increase in HDL-C levels, while TC, TG, LDL-C, ALT, and UA levels decreased. In terms of the differences between the two groups, in terms of TG, there was a highly significant difference between the female MICT and HIIT groups (P < 0.001), with the female HIIT group experiencing an average reduction in TG of 18.91% post-training. A highly significant difference was also observed between the male MICT and HIIT groups (P < 0.001), where the male HIIT group showed an average decrease in TG of 16.39%. These findings suggest that HIIT is notably effective in reducing TG in both genders, especially in females. Regarding LDL-C, only the male HIIT group showed a significant reduction of 22.13% in the average LDL-C value post-training compared to the male MICT group, with a highly significant difference observed between the groups (P < 0.01). These findings align with those of Mc et al.^40^. This suggests that HIIT might have a positive impact on lipid metabolism and cardiovascular health. Notably, the changes in biochemical indicators among obese male college students were higher than those in obese female college students. This may be related to the influence of gender on metabolic adaptation^41–43^. UA, as the end product of purine metabolism, is an important indicator reflecting purine metabolism and renal function^44^. Elevated serum UA levels are closely associated with the occurrence and development of stroke and can promote the onset of vascular inflammation^45^. In this study, participants in both the HIIT and MICT groups showed a significant decrease in UA levels after 8 weeks of training, consistent with the findings of Liu et al.^46^. ALT is one of the key enzymes reflecting liver function, and a meta-analysis reported that compared to moderate-intensity exercise training, HIIT may offer greater benefits in reducing liver fat, especially in lowering ALT levels^47^. Combining the experimental results with related research, an analysis of the reasons for the improvement in lipid metabolism in college students living with obesity through HIIT can be elucidated. Firstly, compared to MICT, the increased release of growth hormone after HIIT may augment post-HIIT fat oxidation^48^. This elevation in lipid oxidation, mediated by growth hormone release, potentially explains why HIIT induces a more significant reduction in lipid-related indicators compared to MICT. Secondly, the dynamic intensity of HIIT, characterized by continuous variation, challenges the body's rapid adaptation, mitigating the likelihood of energy-saving adaptations. Consequently, the body expends more energy, resulting in an elevated lipid metabolism rate^49^. Lastly, due to the propensity for greater EPOC effects in HIIT training, the body maintains a heightened metabolic rate during the post-exercise recovery period. This process relies extensively on lipid-based energy sources, leading to the substantial consumption of fats over an extended period. This phenomenon is conducive to improving lipid metabolism, thereby ameliorating the accumulation of TG and total TC^50^.

Additionally, TC, TG, and HDL-C are indicators linked to insulin resistance and hold predictive value in the occurrence and progression of insulin resistance. Excessive accumulation of fat cells and increased TG levels directly affect glucose metabolism, contributing to insulin resistance. Simultaneously, normal insulin levels in the body influence the activity of lipoprotein lipase. Physical exercise effectively boosts the activity of lipoprotein lipase, underscoring the importance of maintaining normal insulin levels to sustain the enzyme's activity. In conclusion, based on the analysis and discussions above, it can be inferred that long-term, consistent high-intensity interval training effectively ameliorates lipid metabolic disorders in obese populations. This improvement in the body's condition can greatly enhance cardiovascular health by promoting positive changes in lipid profiles.

## Conclusions

HIIT is more effective than MICT in improving body composition and certain biochemical indicators, specifically TC, LDL-C, ALT, UA, and TG, in obese college students. Therefore, HIIT may be considered a preferable exercise intervention for this population to achieve better health outcomes.

## Limitations

This study lacks a non-exercise control group, which restricts our ability to evaluate the impact of exercise interventions compared to no exercise at all. In future research, adding a control group that does not engage in any systematic exercise would allow for a more accurate and scientific assessment of the health benefits of exercise interventions. While participants' maximum heart rates were tested prior to the experiment, these rates could increase over the duration of the training, potentially leading to an increase in exercise intensity. This study did not utilize formal food records, such as food diaries or 24-h recalls, to meticulously track participants' daily food intake, not fully eliminating the potential influence of dietary factors on the study outcomes. We are considering implementing more rigorous dietary control measures in future studies to more accurately assess the effects of exercise interventions. Additionally, further in-depth research is required to validate and reinforce our findings.

## Data Availability

The original contributions presented in the study are included in the article; further inquiries can be directed to the corresponding author.
